# Pulmonary rehabilitation capacity building through a teach-the-teacher programme: A Malaysian experience

**DOI:** 10.7189/jogh.13.03047

**Published:** 2023-08-11

**Authors:** Soo Chin Chan, Hooi Chin Beh, Jayakayatri Jeevajothi Nathan, Yasothai Sahadeevan, Julia Patrick Engkasan, Su Yee Chuah, Eu Way Pek, Nurdiana Abdullah, Chee Kuan Wong, Norita Hussein, Anwar Suhaimi, Nik Sherina Hanafi, Fatim T Mirza, Saari Mohamad Yatim, Hilary Pinnock, Siân William, Ee Ming Khoo, RESPIRE Collaborators

**Affiliations:** 1 Department of Rehabilitation Medicine, Faculty of Medicine, Universiti Malaya, Kuala Lumpur, Malaysia; 2 Department of Primary Care Medicine, Faculty of Medicine, Universiti Malaya, Kuala Lumpur, Malaysia; 3 Department of Medicine, Faculty of Medicine, Universiti Malaya, Kuala Lumpur, Malaysia; 4 Faculty of Health Sciences, University Teknologi MARA (UiTM), Puncak Alam, Selangor, Malaysia; 5 Department of Rehabilitation Medicine, Serdang Hospital, Selangor, Malaysia; 6 NIHR Global Health Research Unit on Respiratory Health (RESPIRE), Usher Institute, University of Edinburgh, Edinburgh, UK; 7 International Primary Care Respiratory Group (IPCRG), Edinburgh, UK

## CHRONIC RESPIRATORY DISEASES AND PULMONARY REHABILITATION

Chronic respiratory diseases (CRDs) are a leading cause of death and hospitalisation in low- and middle-income countries (LMICs) [1], with chronic obstructive pulmonary disease (COPD) being a major contributor to work productivity loss [2]. Pulmonary rehabilitation (PR) is a low-cost therapy that improves symptoms, quality of life, and exercise capacity in patients with a CRD, and can increase productivity [3]. However, there is a lack of a comprehensive PR programme in Malaysia due to inadequate resources and skilled personnel [4]. Strengthening health care workforce capacity is critical to support the development of a local PR faculty with the knowledge and teaching skills to cascade teaching.

## TEACH-THE-TEACHER MODEL

**Figure:**
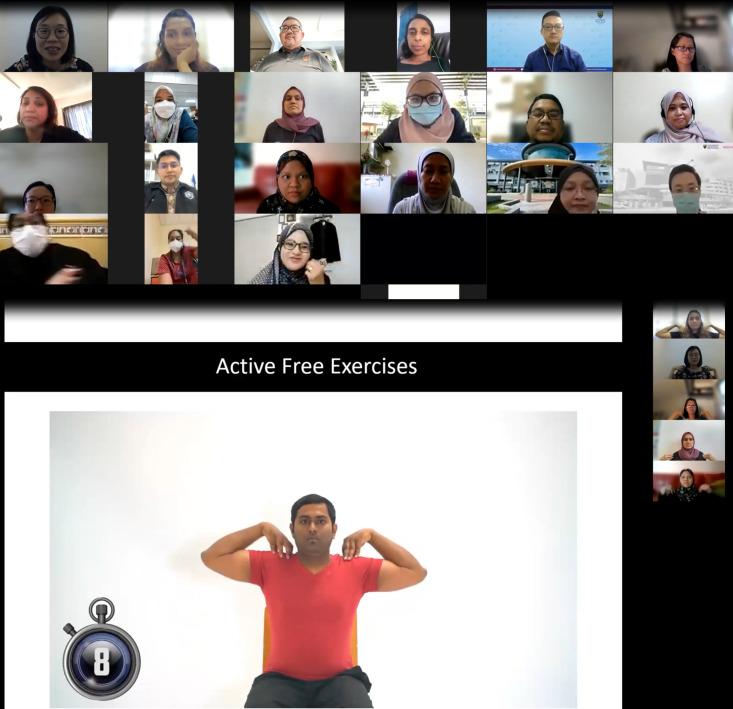
Photo: Screenshot of Zoom meeting taken during the teaching-the-teacher programme. Source: author’s own photo (JJN). All participants featured in this photo have provided their consent for its use.

The teach-the-teacher (TtT) model is an educational approach adapted from the International Primary Care Respiratory Group (IPCRG) that aims to build sustainable local capacity in teaching and health care delivery [5]. It involves an education expert teaching other health care professionals (HCPs) both the subject matter and teaching skills so that they can become teachers and educate other groups of HCPs. A two-day TtT workshop on developing a PR module and programme was held virtually in March 2022, drawing 48 participants, including rehabilitation physicians, chest physicians, palliative care physicians, primary care physicians, physiotherapists, occupational therapists, dieticians, medical social workers, palliative care nurses, and nurse coordinators. The participants were given comprehensive training on designing a structured PR programme and provided the knowledge and skills necessary to anticipate potential challenges in implementing the programme at their health care facilities. To ensure understanding and skill acquisition, the workshop incorporated various teaching methods such as presentations, interactive discussions, case studies, and practical exercises, allowing participants to actively engage with the content, ask questions, and apply their learning in a simulated or real-world context. Their understanding and skill acquisition were assessed through quizzes and knowledge checks, enabling facilitators to gauge the participants' grasp of the concepts and their ability to apply them effectively. The workshop organisers also provided follow-up support for the participants; by offering a feedback form, they allowed them to share their thoughts, suggestions, and concerns about the workshop so as to provide valuable input for future improvements. Moreover, the organisers provided the participants with access to additional learning material so that they could further improve their understanding of the PR module and programme beyond the workshop itself, and advance their education and skill development. Discussions were facilitated through group chats via WhatsApp, where the participants could connect, exchange ideas, and seek advice from one another. These collaborative discussions were designed to enhance the learning experience and foster a sense of community among the participants. The workshop organisers recognised the significance of individualised assistance in effectively implementing the PR module and programme, so they offered mentoring opportunities, personalised guidance, and support. Experienced professionals took on the role of mentors, offering valuable guidance, answering questions, and providing advice based on their own expertise, which further empowered participants in their professional development and successful implementation of the PR programme.

## STEPS IN DESIGNING A PR TEACHING PROGRAMME

### Identify target population

The success of the TtT programme depends on identifying the appropriate target population. With this in mind, the learning needs of the population were assessed, competencies mapped, and relevant groups prioritised. These groups included rehabilitation physicians, respiratory physicians, primary care physicians, staff nurses, therapists, medical social workers, pharmacists, and dieticians. The head of service and target population were identified as key in ensuring teaching was cascaded.

### Setting learning objectives

The TtT workshop taught participants how to create clear learning objectives appropriate for their diverse learning contexts through interactive lectures. The specific, measurable, achievable, relevant, and time-bound (SMART) was used to define realistic targets [6], while the audience-behaviour-condition-degree (A-B-C-D) was introduced to create learning objective, such as the following: “After completing this TtT programme, you (“Audience”) should be able to develop (“Behavior”) an algorithm (“Degree”) to assist PR services (“Condition”) in being upscaled across the country within resource constraints”.

### Designing a programme

Workshop participants learned how to create a teaching programme or content by identifying the components of learning outcomes such as knowledge, skills, or attitude, which should align with the learning objectives. They were explained the fundamental ideas of teaching approaches such as physical lectures, hands-on workshops, working with small groups, distance learning, and webinars, and coached on how to design instructional content and identify teaching approaches during the workshop.

### Preparing and using teaching materials

Effective engagement and good presentation delivery require proper selection of teaching methods and resources. Passive teaching methods have lower learning retention rates compared to participation teaching methods [7]. Participants in the TtT workshop were able to select adaptable tools based on learning objectives, target groups, classroom size, and distance learning [8], and were taught to choose the ideal teaching approach for their programme, such as group activities or projects, individual independent work, role plays, demonstrations, or case studies [8].

### Assessing learning needs

During the TtT workshop, participants were introduced to various types of assessments and taught how to distinguish between objective and subjective assessments. They learned how to plan and evaluate a project and its impact, with the goal of determining mastery of critical skills and knowledge and evaluating the program’s effectiveness over time. To enhance interaction and brainstorming, they were divided into groups, where they discussed practical and theoretical evaluation methods, including direct observation during clinical practice, logbooks, practical assessments, multiple-choice questions, and theoretical quizzes.

### Presentation skills

The success of a presentation heavily relies on the presenter's skill and ability to reach the audience [9]. The SENSES approach was used to deliver the lecture, which includes starting with a story, teaching only essentials, creating a need for participants, stimulating curiosity, providing good examples, and teaching a skill. Participants were engaged through interactive discussions and group work. The learning-by-teaching effect was discussed, as evidence shows that those who teach others demonstrate greater understanding and knowledge [10]. If adopted by other institutions, the TtT workshop can create a group of trained professionals to fill the PR gap for patients with CRDs.

### Developing a structured PR programme

The lack of a systematic PR programme in Malaysia requires the development of a tailored PR model that will work in a particular setting. Interactive lectures and group discussions were conducted to teach participants how to develop a PR programme in their facilities; they were presented with various PR models used globally, such as inpatient PR, outpatient PR, home PR, and community PR, and they agreed on the model that would work best for their facility. A team of medical professionals, including physicians, nurses, therapists, social workers, counsellors, pharmacists, dieticians, and palliative care, is required to establish a PR programme. The participants discussed the target population for PR, including individuals with CRD post-COVID-19, interstitial lung disease, COPD, bronchiectasis, and post-lung surgery. Inpatient PR could take place in the respiratory ward, while outpatient PR could be held in the rehabilitation department therapy area. Resources such as educational materials, a resistive band, a QR code for an exercise video, and an incentive spirometer are necessary. The programme should include COPD education, coping strategies, energy conservation, activities of daily living (ADL) retraining, assistive devices, pharmacological optimisation, nutrition, smoking cessation, self-management, end-of-life discussion, and assessments. The programme should also include St George's Respiratory Questionnaire (SGRQ-C), a quality of life (QOL) questionnaire, and an exercise component-breathing exercises, resistance exercise, endurance training, and a home exercise programme. Assessments should include the two-minute walking test (2MWT) for inpatients and the six-minute walking test (6MWT) and spirometry for outpatients. The programme should use the Donabedian framework to evaluate care quality, with a multidisciplinary team involved in general exercise, functional activities, psycho-social therapies, education, and specific actions tailored to patients' priorities and goals. Comprehensive assessments would include various tests and questionnaires. The participants plan to establish a PR programme in their facility soon, and policymakers' involvement is crucial to advance PR uptake in the country. To achieve this, they suggest introducing the programme at the hospital level, encouraging early referrals, utilising pre-existing PR services, incorporating clinical practice guidelines, providing more teaching programmes, and involving primary care physicians in health clinics to develop a community PR programme.

### Challenges in implementing PR progamme

Following the training, HCPs engaged in conversations regarding potential challenges they may encounter when initiating a PR programme at their respective facilities, including Malaysia (**Table 1**).

**Table 1:** Potential challenges and solutions in the implementation of PR programme in Malaysia

Challenges	Solutions
Low knowledge, skills, and awareness of PR among HCPs	HCPs play a transdisciplinary role and recommending to policy makers that PR services be included as a component under the health ministry’s domiciliary care programme
	Formal PR teaching of HCPs, appointment of motivated programme coordinators with expert knowledge of PR,
	expanding PR programmes, promoting PR to primary teams that includes respiratory physicians and primary care physicians to increase referrals rates, developing a referral pathway for primary teams to refer for PR, and developing national PR guidelines
Human resources, logistics and accessibility	Teach a champion or leader in the community to lead or guide the whole community or population in a designated area/location/village/community
Lack of a structured PR programme	Provide written guidelines, protocols and streamlined referral pathways as well as TtT PR programme
Patient participation barriers which included patients in critical or end-stage of the disease, and compliance in completing the programme as well as patients’ and carers’ low knowledge and awareness about end-stage CRDs	Community outreach programmes with hospital-community collaboration, patient education and awareness about PR, HCPs working with patients to understand their needs and expectations regarding PR
	Early intervention/referral by introducing PR programmes to improve patients' understanding, caregiver empowerment by assisting in convincing patients of the benefits of PR programmes, and patient empowerment by reminding them that their condition can be improved with a better quality of life
Lack of funding (to acquire new equipment or replace malfunctioning equipment)	Look for external funding or government assistance

PR – pulmonary rehabilitation, HCPs – health care professionals, TtT – teacher the teacher

### Limitations and strengths in teach-the-teacher workshop

The workshop was originally intended to include a visit to the country's top PR facilities, but due to the rapid spread of the Omicron strain of COVID-19, it adopted a virtual format and the site visit was cancelled. This visit was expected to provide participants with a better understanding of the components of a well-structured PR programme. However, the virtual format allowed HCPs from remote locations to participate without travel restrictions.

## CONCLUSIONS

In Malaysia, the development of a well-structured PR programme remains a challenge, which could be resolved through a TtT programme, which could train more HCPs to become teachers and expand PR services across the country. The workshop participants identified potential challenges in the development of PR and proposed solutions to overcome them. By addressing these concerns, HCPs could efficiently create and implement PR programmes at their facilities.

## References

[1] World Health Organization. The top 10 causes of death. 2020. Available: https://www.who.int/news-room/fact-sheets/detail/the-top-10-causes-of-death. Accessed: 25 March 2022.

[2] Ur Rehman A Hassali MAA Muhammad SA Shakeel S Chin OS Ali IABH Economic Burden of Chronic Obstructive Pulmonary Disease Patients in Malaysia: A Longitudinal Study. PharmacoEconomics Open. 2021;5:35-44. 10.1007/s41669-020-00214-x 32291727 PMC7895885

[3] Holland AE Cox NS Houchen-Wolloff L Rochester CL Garvey C ZuWallack R Defining Modern Pulmonary Rehabilitation. An Official American Thoracic Society Workshop Report. Ann Am Thorac Soc. 2021;18:e12-29. 10.1513/AnnalsATS.202102-146ST 33929307 PMC8086532

[4] Ministry of Health Malaysia. Clinical Practice Guidelines Management of Chronic Obstructive Pulmonary Disease. Putrajaya: Ministry of Health Malaysia; 2009.

[5] International Primary Care Respiratory Group. IPCRG Teach the Teacher: teaching how to teach, adapt, think and do. 2021. Available: https://www.ipcrg.org/projects/education/teach-the-teacher. Accessed: 25 March 2022.

[6] Doran GT There’s a S.M.A.R.T. way to write management’s goals and objectives. Management Review. 1981;70:35-6.

[7] Northwest Centre for Public Health Practice. Effective Adult Learning. A toolkit for teaching adults. 2012. Available: https://www.nwcphp.org/training/effective-adult-learning-a-toolkit-for-teaching-adults. Accessed: 25 March 2022.

[8] Cantillon P, Hutchinson L, Wood D. ABC of Learning and Teaching in Medicine. London: BMJ Publishing Group; 2003.

[9] Gelula MH Effective lecture presentation skills. Surg Neurol. 1997;47:201-4. 10.1016/S0090-3019(96)00344-8 9040825

[10] Newble D, Cannon R. A Handbook for Medical Teachers 4th ed. New York: Kluwer Academic Publishers; 2002.

